# ILViT: An Inception-Linear Attention-Based Lightweight Vision Transformer for Microscopic Cell Classification

**DOI:** 10.3390/jimaging11070219

**Published:** 2025-07-01

**Authors:** Zhangda Liu, Panpan Wu, Ziping Zhao, Hengyong Yu

**Affiliations:** 1 College of Computer and Information Engineering, Tianjin Normal University, Tianjin 300387, China; zhangdaliu@stu.tjnu.edu.cn (Z.L.); ztianjin@126.com (Z.Z.); 2 Department of Electrical and Computer Engineering, University of Massachusetts Lowell, Lowell, MA 01854, USA

**Keywords:** cell classification, linear attention, inception architecture

## Abstract

Microscopic cell classification is a fundamental challenge in both clinical diagnosis and biological research. However, existing methods still struggle with the complexity and morphological diversity of cellular images, leading to limited accuracy or high computational costs. To overcome these constraints, we propose an efficient classification method that balances strong feature representation with a lightweight design. Specifically, an Inception-Linear Attention-based Lightweight Vision Transformer (ILViT) model is developed for microscopic cell classification. The ILViT integrates two innovative modules: Dynamic Inception Convolution (DIC) and Contrastive Omni-Kolmogorov Attention (COKA). DIC combines dynamic and Inception-style convolutions to replace large kernels with fewer parameters. COKA integrates Omni-Dimensional Dynamic Convolution (ODC), linear attention, and a Kolmogorov-Arnold Network(KAN) structure to enhance feature learning and model interpretability. With only 1.91 GFLOPs and 8.98 million parameters, ILViT achieves high efficiency. Extensive experiments on four public datasets are conducted to validate the effectiveness of the proposed method. It achieves an accuracy of 97.185% on BioMediTech dataset for classifying retinal pigment epithelial cells, 97.436% on ICPR-HEp-2 dataset for diagnosing autoimmune disorders via HEp-2 cell classification, 90.528% on Hematological Malignancy Bone Marrow Cytology Expert Annotation dataset for categorizing bone marrow cells, and 99.758% on a white blood cell dataset for distinguishing leukocyte subtypes. These results show that ILViT outperforms the state-of-the-art models in both accuracy and efficiency, demonstrating strong generalizability and practical potential for cell image classification.

## 1. Introduction

Accurate cell classification plays a critical role in supporting clinical decision-making [1]. However, traditional classification methods face significant challenges when handling complex and high-dimensional microscopic cell images, and most of the existing approaches rely on conventional machine learning algorithms or convolutional neural networks (CNNs). They are deep learning models designed for automatically learn complex patterns through hierarchical feature learning from an image. For example, Das et al. [2] employed several machine learning techniques, including random forests (an ensemble method based on multiple decision trees), gradient boosting (an iterative ensemble technique that incrementally adds models to correct previous errors), k-nearest neighbors (a non-parametric algorithm that classifies samples based on the majority category of the k closest instances in the feature space), and support vector machines (SVMs, a supervised learning algorithm that identifies the optimal hyperplane by maximizing the margin between classes), alongside the deep learning model ResNet-50 to perform five-class classification of cervical squamous epithelial cells using the SIPaKMeD dataset. Similarly, Priyadarsini et al. [3] proposed an improved CNN-SVM framework that combines region-based CNN feature extraction and object detection to improve the diagnosis accuracy of cervical cancer. While these methods have achieved moderate success, they still exhibit limitations in feature representation, particularly in complex environments and when dealing with highly variable cell morphologies.

In recent years, self-attention mechanisms have shown promising results in image classification tasks, with hybrid convolution-attention architectures emerging as a popular direction in cell image analysis. For example, Inturu et al. [4] proposed a white blood cell classification framework that combines EfficientNet with the Vision Transformer (ViT). EfficientNet achieves a balance between accuracy and efficiency through its compound scaling method and a neural architecture search-based efficient baseline network. ViT employs a pure Transformer architecture for image classification by decomposing images into serialized patches and processing them through a standard Transformer encoder, demonstrating strong scalability and generalizability with excellent performance during large-scale pretraining. The integration of EfficientNet and ViT leads to improved classification accuracy. Wan et al. [5] introduced a hierarchical framework based on multi-scale local binary convolutional networks, demonstrating accurate classification of cervical cell images. Despite their excellent performance, they often incur high computational costs, making them less suitable for deployment in a resource-constrained environment, a common scenario in real-world cell classification applications.

To address the aforementioned limitations, we propose a novel classification method, Inception-Linear Attention based Lightweight Vision Transformer (ILViT). The ILViT model integrates two key modules: Dynamic Inception Convolution (DIC) and Contrastive Omni-Kolmogorov Attention (COKA). The DIC module improves the representation of features by using dynamic convolution [6] and an Inception-style convolutional structure [7]. The COKA module, designed for lightweight global feature extraction, incorporates omni-dimensional dynamic convolution for adaptive kernel weighting [8], contrastive normalization to alleviate dimensional collapse [9], a linear attention mechanism optimized via activation functions, and the KAN structure inspired by the Kolmogorov–Arnold representation theorem that establishes that any continuous multivariable function can be represented as a superposition of continuous univariate functions [10,11]. By combining these modules, the proposed ILViT model achieves improved classification accuracy while significantly reducing computational overhead. While the current study specifically validates ILViT’s effectiveness in microscopic cell classification, we believe that the architecture’s core design principles possess inherent transferability. ILViT could be adapted for other vision tasks.

The main contributions of this paper are threefold. First, we design a novel DIC module by synergistically combining dynamic convolution and an Inception-style structure, effectively replacing large-kernel convolutions while maintaining wide receptive field with fewer learnable parameters. Second, we propose a COKA module that integrates omni-dimensional dynamic convolution, enhanced linear attention, and the KAN structure, enabling powerful feature representation capabilities with exceptional computational efficiency. Third, we propose a lightweight framework ILViT for microscopic cell classification with quantization-optimized linear layers and enhanced activation functions. ILViT overcomes key limitations of conventional methods and establishes a new paradigm for accurate and resource-efficient cellular image analysis.

## 2. Related Work

Microscopic cell image classification has been widely applied in the biomedical image analysis domain. Early approaches relied primarily on manual feature extraction and traditional machine learning algorithms. For example, Chen et al. [12] integrated single-cell and bulk transcriptomic data and applied CIBERSORTx for deconvolution analysis to reveal alterations in cellular composition associated with endometriosis. A random forest–based early prediction model was constructed to distinguish between endometriosis patients and healthy controls. However, these methods often encounter performance bottlenecks when applied to complex image data.

In recent years, CNNs have significantly advanced the field of cell classification. Chossegros et al. [13] employed the EfficientNet model to combine limited sample fine-tuning and color transformation to improve generalization in blood cell classification tasks. Mpofu et al. [14] introduced a quantum convolutional neural network (QCNN) to distinguish malarial and non-malarial cells. Furthermore, the adoption of transfer learning has enabled CNN-based models to maintain high accuracy even with limited training data. For example, Daas et al. [15] leveraged pretrained CNNs to enhance leukemia detection performance.

Despite the success of deep learning in cell image classification, several challenges remain. Due to the inherent heterogeneity and morphological variability of cell images, deep learning-based models are prone to overfitting or inadequate feature representation [16]. In addition, factors such as varying image resolutions, illumination inconsistencies, and noise may adversely affect model performance. It remains an open challenge to improve the robustness and stability of classification models in different imaging conditions [17]. Furthermore, cell images typically exhibit complex structural and spatial patterns. Traditional deep learning methods often emphasize local features, overlooking global structures and spatial dependencies within the image.

To overcome these limitations, recent studies have incorporated spatial information using attention mechanisms. Halder et al. [18] fine-tuned a pretrained Vision Transformer (ViT) model [19] in the MedMNISTv2 dataset to evaluate its classification performance in different biomedical image modalities. Such methods enhance the model’s ability to distinguish subtle morphological differences, especially across cell subtypes or in cases of cellular transformation. However, attention-based models often suffer from high computational costs and large parameter sizes, rendering them unsuitable for deployment in large-scale or resource-constrained scenarios.

In response to the aforementioned challenges, we propose a novel approach to address the limitations of existing models. The proposed method aims to improve classification accuracy and robustness while maintaining a lightweight architecture, delivering superior performance while maintaining practical resource constraints.

## 3. Methodology

### 3.1. Overall Architecture

**Figure 1:**
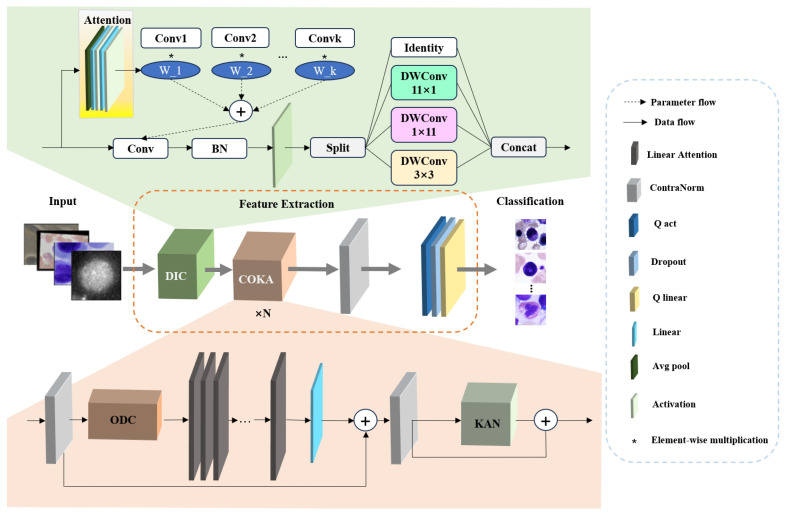
Architecture overview of the proposed ILViT.

The architecture overview of the proposed ILViT method is illustrated in Figure 1. The baseline model is MViTv2. MViTv2 provides an efficient and powerful visual modeling architecture for a variety of visual tasks by designing a multi-scale feature hierarchy, an improved Pooling Attention, and a hybrid window attention mechanism. Although MViTv2 has excellent visual modeling capabilities, its architectural components, particularly large-kernel convolutions and attention modules, impose constraints on both parameter count and feature extraction capacity. In this paper, modular improvements are introduced to overcome these challenges. The model comprises three major stages: input preprocessing, feature extraction, and final classification. Initially, the input microscopic image is passed through the DIC module. In this module, dynamic convolution is applied, where *K* weights are learned to process *k* identical convolution kernels, enabling adaptive learning of input-specific features. Following this, the input is processed by an Inception-style convolutional structure composed of four parallel branches. This design improves computational efficiency while preserving feature diversity and representation capability. The outputs from the four branches are concatenated and fed into the COKA module. The COKA module is stacked *N* times to ensure sufficient feature learning and hierarchical abstraction. Within each COKA block, a normalization layer is first applied to reduce the internal covariate shift and mitigate overfitting. Subsequently, the Omni-Dimensional Dynamic Convolution (ODC) is employed to compensate for the limitations of earlier dynamic convolutions by adaptively adjusting kernel weights across four dimensions, thereby maximizing the representational capacity of convolutional layers. Next, the feature map is passed to the linear attention mechanism, which is enhanced by activation functions to effectively capture global contextual information. Another normalization layer is applied after attention to stabilize gradients and improve feature quality. At the end of each COKA module, a Kolmogorov–Arnold Network (KAN) structure is incorporated to introduce strong nonlinearity and further augment the model’s representational power. Finally, an additional normalization layer is employed at the end of this method to improve model generalization, followed by quantized activation functions and quantized linear layers, both of which contribute to efficient inference and lightweight deployment.

### 3.2. Dynamic Inception Convolution (DIC)

As illustrated in Figure 1, the DIC module is primarily composed of a dynamic convolution mechanism and an Inception-style convolutional block. The dynamic convolution improves the model’s representational capacity by aggregating multiple parallel convolutional kernels in an adaptive manner within each convolutional layer. This allows the network to dynamically adjust its receptive fields and kernel responses based on input features. The Inception module within DIC is designed to address computational efficiency by employing four parallel branches: a small square kernel, two orthogonal asymmetric kernels, and an identity mapping. This architectural choice significantly reduces the number of learnable parameters and FLOPs, thus improving overall computational efficiency without sacrificing model accuracy. The pseudo-code is shown in Algorithm 1. The detailed procedures is as follows.

The input feature map is first divided into four parallel branches:

(1) $$X_{hw},X_{w},X_{h},X_{id}=Split\left(X\right).$$

Each branch is processed separately by a different convolutional kernel, calculated as:

(2) $${X_{hw}}^{\prime }={DWConv}_{ks\times ks}\left(X_{hw}\right),$$

(3) $${X_{w}}^{\prime }={DWConv}_{1\times kb}\left(X_{w}\right),$$

(4) $${X_{h}}^{\prime }={DWConv}_{kb\times 1}\left(X_{h}\right),$$

(5) $${X_{id}}^{\prime }=X_{id},$$

where ks is the size of the small square kernel and defaults to 3 and kb is the size of the band kernel and defaults to 11. Finally, the outputs of the four branches are spliced to obtain the output feature map

(6) $$X^{\prime }=\mathrm{Concat}\left({X_{hw}}^{\prime },{X_{w}}^{\prime },{X_{h}}^{\prime },{X_{id}}^{\prime }\right).$$

**Algorithm 1** DIC**Require:** $x\in R^{\left(H\times W\times C\right)}$ **Ensure:** *y*
 1: x=FC2(FC1(avgpool(x)))
 2: Projection_x=Conv2d(softmax(x))
 3: x_hw,x_w,x_h=split(Projection_x)
 4: $x\_{hw}^{\prime }=\mathrm{dwconv}\left(x\_hw\right)$,$x\_w^{\prime }=\mathrm{dwconv}\left(x\_w\right)$,$x\_h^{\prime }=\mathrm{dwconv}\left(x\_h\right)$
 5: $y=\mathrm{concat}(x\_{hw}^{\prime },x\_w^{\prime },x\_h^{\prime })$


### 3.3. Contrastive Omni-Kolmogorov Attention (COKA)

**Figure 2:**
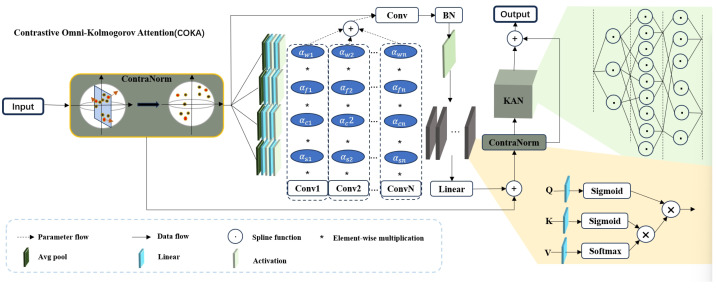
Illustration of COKA module structure.

Figure 2 shows more details of the COKA module. It comprises a contrastive normalization layer, ODC, a linear attention layer, and the KAN module. The contrastive normalization layer, motivated by contrastive learning, disperses feature representations in the embedding space to alleviate dimensional collapse. ODC captures information along four dimensions of the convolutional kernel, offering finer-grained attention allocation than standard dynamic convolution. Its formulation is as follows:

(7) $$y=(\alpha_{w1}*\alpha_{f1}*\alpha_{c1}*\alpha_{s1}*W_{1}+\ldots +\alpha_{wn}*\alpha_{fn}*\alpha_{cn}*\alpha_{sn}*W_{n})*x,$$

where *x* and *y* represent the input and output of ODC, respectively, and ‘∗’ represents the convolution operation. Compared to conventional self-attention mechanisms [20], linear attention layers significantly reduce both the number of parameters and computational complexity. By integrating activation functions into the representation of queries (*Q*), keys (*K*), and values (*V*), the model can improve the allocation of feature importance and alter the computation sequence of QKV. This adjustment leads to a substantial reduction in computational cost without compromising performance. The specific formulation is as follows:

(8) $$\mathrm{LinearAttention}\left(Q,K,V\right)=\frac{\mathrm{Sigmoid}\left(Q\right)(\mathrm{Sigmoid}\left(K^{T}\right)\mathrm{Softmax}\left(V\right))}{\sqrt{d_{k}}},$$

where $d_{k}$ denotes the dimensionality of the data.

The final component of the COKA module is the KAN structure which serves as a replacement for the traditional multilayer perceptron (MLP). This structure is specifically designed to overcome MLP limitations. KAN achieves superior nonlinear representation capacity with significantly fewer trainable parameters. Unlike MLPs that use fixed activation functions (e.g., ReLU, Sigmoid) at nodes and rely on linear weights, KAN implements trainable univariate functions parameterized as splines along network edges. This design not only provides greater flexibility and adaptability in learning complex functions but also eliminates the need for linear transformations, directly focusing on the functional relationships between inputs and outputs. By replacing linear weights with spline-parameterized univariate functions, KAN achieves representational performance comparable to that of MLPs in a significantly more lightweight and computationally efficient manner. This approach reduces the number of trainable parameters. Moreover, the learnable activation functions on edges and the absence of linear weights make KANs more interpretable than MLPs. This improved interpretability is crucial for applications in science and engineering, where understanding the model’s behavior is as important as its performance. Additionally, KANs can avoid catastrophic forgetting, a common issue in MLPs, by leveraging the locality of spline parametrizations. This property makes KANs more suitable for continual learning tasks, where the model needs to adapt to new data without forgetting previously learned information. Overall, the KAN structure offers a more efficient, flexible, and interpretable alternative to MLPs, making it a valuable component in the COKA module for various applications. The pseudo-code of COKA is shown in Algorithms 2 and 3.
**Algorithm 2** ODC
1: **Input:**
$x\in R^{\left(H\times W\times C\right)}$
2: **Output:**
*y*
3: x=Conv2d(ReLu(Conv2d(avgpool(x))))
4: Attention_weights=FC2(ReLu(FC1(Global_Average_Pooling(x))))
5: Attention_values=Sigmoid(Attention_weights)
6: **for**
i = 1
**to**
*n*
**do**
7:  W_i_weighted=Attention_values[:,i:i+1]×W_i
8: **end for**
9: $W\_\mathrm{combined}=\sum_{i=1}^{n}W_{i}\_\mathrm{weighted}$
10: y=Conv2d(x,W_combined)


**Algorithm 3** COKA
1: **Input:**
$x\in R^{\left(H\times W\times C\right)}$
2: **Output:**
*y*
3: $x_{1}=x$
4: x_norm=ContraNorm(x)
5: x_conv=ODC(x_norm)
6: **Parallel heads:**
7: $Q,K,V=\mathrm{Linear}(x\_{\mathrm{conv}}_{1},x\_{\mathrm{conv}}_{2},x\_{\mathrm{conv}}_{3})$
8: $\mathrm{Linear}\_\mathrm{Attn}\left(Q,K,V\right)=\frac{\mathrm{Sigmoid}\left(Q\right)(\mathrm{Sigmoid}\left(K^{T}\right)\mathrm{Softmax}\left(V\right))}{\sqrt{d_{k}}}$
9: x_concat=Concatenate(Parallelheads)
10: $x^{\prime }=x_{1}+x_{\mathrm{concat}}$
11: $x^{\prime \prime }=x^{\prime }$
12: $x_{\mathrm{kan}}=\mathrm{KAN}\left(\mathrm{ContraNorm}\left(x^{\prime }\right)\right)$
13: $y=x_{\mathrm{kan}}+x^{\prime \prime }$


### 3.4. Quantitative Operation

In our model, certain activation functions and fully connected layers involve quantization operations, primarily inspired by FQ-ViT [21]. The QAct layer accelerates inference while preserving model performance by combining log2-based quantization with a polynomial approximation of the exponential function [22]. In addition to quantized activation functions, quantized linear layers are also employed. Specifically, in the final layer of the ILViT method, a uniform quantization strategy is applied to optimize the linear layer [23,24].

### 3.5. Loss Function

The loss function employed in this method is the Cross-Entropy Loss. As a widely used objective function for classification tasks, the cross-entropy loss measures the divergence between the predicted probability distribution and the true label distribution. It penalizes predictions that deviate from the ground truth, guiding the model to produce more accurate classifications. The mathematical formulation is given as

(9) $$\mathrm{CrossEntropyLoss}=-\sum_{i=1}^{C}y_{i}\log\left(p_{i}\right),$$

where *C* represents the number of classes in the classification task, $y_{i}$ denotes the distribution of the true labels (1 for the correct class and 0 for all other classes), and $p_{i}$ refers to the predicted probability that is the likelihood that the sample belongs to class *i*.

## 4. Experiments and Results

### 4.1. Dataset

To validate the effectiveness of the proposed ILViT method, four datasets are utilized. The details of these datasets are provided in Table 1.

(1) BioMediTech Dataset: The BioMediTech dataset contains 195 original high-resolution super-images of retinal pigment epithelial cells at different stages, captured by using a microscope. Each super-image is divided into a 4 × 4 grid, resulting in 16 images. After removing those images that are noisy, blurred, or contained only background, we have 1862 valid images. All images are labeled with categories by two professional annotators and used for evaluation.
(2) ICPR-HEp-2 Dataset: The HEp-2 cells in the ICPR-HEp-2 dataset express a variety of nuclear antigens, making them ideal for indirect immunofluorescence (IIF) experiments. This dataset, provided by the University of Salerno, includes fluorescence microscopy images of HEp-2 cells with different morphological forms.
(3) Hematological Malignant Tumor Bone Marrow Cell Dataset: This dataset consists of bone marrow smears from 945 patients, including more than 170,000 cells that have been annotated and anonymized by experts. The smears are stained using the May-Grünwald-Giemsa/Pappenheim method and captured with a 40× oil immersion objective. The dataset includes cells from 21 different categories, but due to the imbalance in data distribution, six cell types are selected for experiments.
(4) Leukocyte Classification Dataset: The Leukocyte Classification dataset comprises 12,500 blood cell images that have been augmented, and labeled according to its respective cell type. In the diagnosis of blood-related diseases, the identification and extraction of features from blood samples is a critical step. Therefore, it is of significant importance to develop automated methods for detection and classification of blood cell subtypes.

The datasets are divided into training and testing sets in the ratio of 8:2. For the training set, images are first rotated randomly between −15 to 15 degrees, then multiple image enhancement operations using the “AutoAugmentPolicy.IMAGENET” [29] are applied to improve the robustness of the model to different image variations, and finally they are resized to 224 × 224 and normalized with mean values of [0.485, 0.456, 0.406] and standard deviations of [0.229, 0.224, 0.225].

### 4.2. Evaluation Metrics

In the experiments, the scikit-learn package is used to compute three evaluation metrics: accuracy, recall, and F1 score. Under the micro-average mode, because both recall and F1 score yield the same results as accuracy, we primarily focus on accuracy for model evaluation. The formula for calculating accuracy is as follow:

(10) $$\mathrm{Accuracy}=\frac{\sum_{i=1}^{C}TP_{i}}{\sum_{i=1}^{C}\left(TP_{i}+FP_{i}+FN_{i}\right)},$$

where *C* represents the total number of classes, and $TP_{i}$, $FP_{i}$, and $FN_{i}$ denote the true positives, false positives, and false negatives for the *i*-th class, respectively.

### 4.3. Comparative Experiments

#### 4.3.1. Experimental Setup

The proposed method is evaluated on four datasets by comparing with several representative state-of-the-art image classification models, including ResNet [30], RegNet [31], ShuffleNetV2 [32], Vision Transformer (ViT) [19], Swin Transformer [33], ConvNeXt [34], and MViTv2 [35]. All models are trained for 500 epochs to ensure a fair comparison. The hyperparameter configurations used in the experiment are listed in Table 2. The overall architectures of these comparative models are provided in Table 3.

#### 4.3.2. Results and Analysis

The quantitative results are summarized in Table 4. Our findings demonstrate that the proposed model consistently outperforms state-of-the-art methods in terms of classification accuracy. As shown in Table 4, the proposed model achieves an accuracy improvement of about 7% over the baseline on the BioMediTech dataset, and notably, it surpasses the classical ShuffleNet model by around 10%, indicating a substantial performance gain. Furthermore, the proposed model achieves an accuracy of 99.75% in the leukocyte classification dataset. These results can be largely attributed to the integration of the proposed DIC and COKA modules, which significantly enhanced feature representation and learning capacity.

#### 4.3.3. Interpretability Analysis

To enhance the interpretability and reliability of the proposed classification method, Gradient-weighted Class Activation Mapping (Grad-CAM) [36] is used to visualize the regions of interest that the model focuses during decision-making. This analysis is conducted on both the BioMediTech and BloodCell datasets. The resolutions of the class activation maps for all models are the same size 224 × 224.

**Figure 3:**
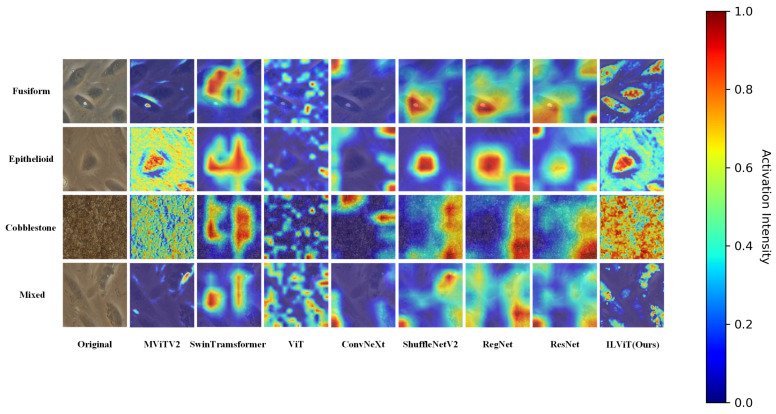
Characteristic plot of different models on the BioMediTech dataset.

As shown in Figure 3, the proposed ILViT model exhibits superior attention visualization compared to other mainstream models. It clearly highlights the contours and distinctive features of the target cells. For example, in the Fusiform category, only ILViT successfully captures the fine structural details of the cells. In the Cobblestone category, the ILViT demonstrates stronger attention to the compact arrangement of cells, with a noticeably higher focus intensity than the baseline. These results indicate that the proposed model has a more robust feature learning capability.

**Figure 4:**
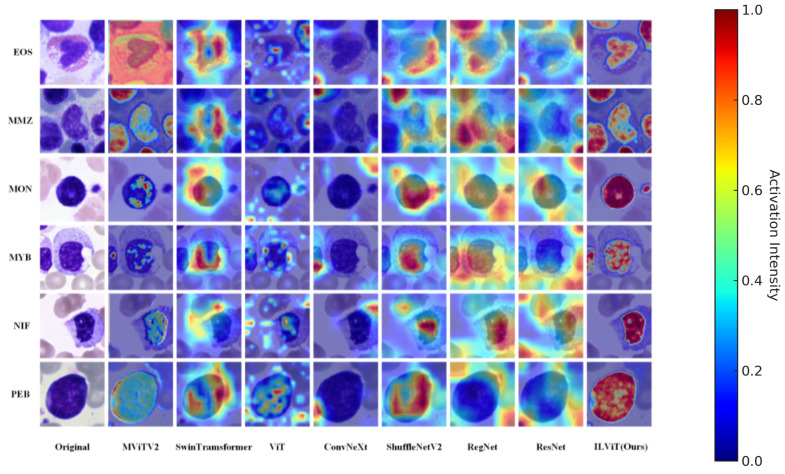
Characteristic plot of different models in the annotated dataset of myeloid cytology experts for selected hematological malignancies.

Figure 4 presents the attention visualizations of different models in the bone marrow cell dataset. Overall, the proposed ILViT model demonstrates a stronger ability to accurately focus on critical regions while effectively suppressing background noise in all categories. For example, in the EOS category, models such as ConvNeXt and Swin Transformer primarily respond to surrounding noise, whereas the ILViT model accurately localizes the key cellular regions. Similarly, in the PEB category, the ILViT model concentrates on cell edges and morphological features, while models like ResNet and MViTv2 show dispersed attention over irrelevant background areas. The ILViT model consistently exhibits clearer boundaries and higher region concentration, emphasizing essential cell regions while suppressing non-informative areas. This advantage enhances both the interpretability and robustness of the model in cell classification tasks.

### 4.4. Ablation Study

#### 4.4.1. Experimental Setup

To evaluate the effectiveness of each component within the proposed framework, an ablation study is conducted on the BioMediTech dataset. Five progressive versions of the model, denoted as ILViT_V0 to ILViT_V4, are developed, with each version introducing incremental architectural improvements over the previous one. All models are trained for 500 epochs to ensure consistency and fair comparison.

- ILViT_V0: Replaces the large-kernel convolution at the front end of the MViTv2 architecture with the proposed DIC module.
- ILViT_V1: Integrates activation functions into the multi-head self-attention mechanism, forming a linear attention structure.
- ILViT_V2: Substitutes the convolutional layer preceding the linear attention module with Omni-dimension dynamic convolution.
- ILViT_V3: Optimizes the normalization layers before and after linear attention, as well as at the end of the model, using a contrastive learning strategy.
- ILViT_V4: Replaces the MLP block following linear attention with a KAN structure and applies quantization to the activation functions and linear layers in the outer framework.

To evaluate model efficiency, each version is fed a standardized input tensor of size 3 × 224 × 224. The lightweight characteristics of each model at a depth of 10 layers are summarized in Table 5, in terms of both computational complexity and parameter count.

#### 4.4.2. Results and Analysis

The ablation results are summarized in Table 5, detailing the classification accuracy and model efficiency in different versions. In ILViT_V0, the introduction of the DIC module leads to an approximate 4% improvement (p<0.05) in accuracy over the baseline, while slightly reducing both computational complexity and parameter count. This efficiency gain is attributed to the decomposition of large-kernel convolutions via the Inception-based DIC module, which maintains performance while reducing resource demands. ILViT_V1 further improves accuracy by approximately 1.5% (p<0.05) over V0 through the integration of the newly designed linear attention mechanism, which also contributes to additional model simplification. In ILViT_V2, Omni-dimension dynamic convolution is applied to the front end of the linear attention module. Although this modification increases computational cost and parameter count due to the additional kernel dimensions, it yields a 0.7% accuracy gain (p<0.05) compared to V1. In ILViT_V3, contrastive normalization is introduced before and after the linear attention module, as well as at the output stage. This change improves accuracy by 0.2% (p<0.05) over V2, without increasing model size or complexity. Finally, ILViT_V4 combines partial quantization with a KAN structure to further enhance both accuracy and efficiency. Compared to V3, V4 achieves a 0.4% improvement (p<0.05) in accuracy, while reducing FLOPs by 54.7% and parameters by 64%. These results demonstrate that the final model effectively balances lightweight design and high performance.

### 4.5. Class Imbalance Analysis

**Figure 5:**
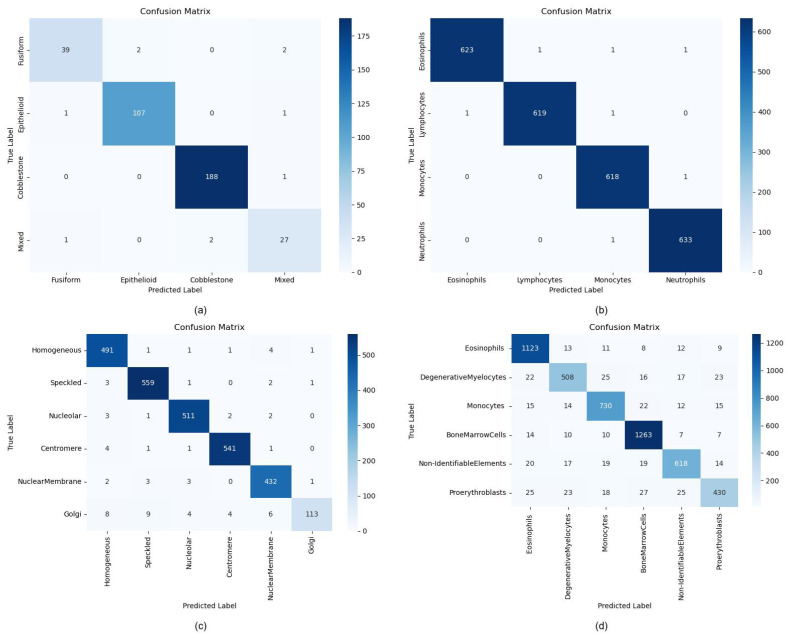
Confusion matrix for different datasets. (**a**) BioMediTech dataset, (**b**) White blood cell dataset, (**c**) ICPR-HEp-2 dataset, (**d**) BoneMarrow dataset.

To investigate the potential impact of class imbalance on classification performance, we generate confusion matrices for each dataset, as shown in Figure 5 and compute the class-specific metrics (accuracy, precision, and recall) recorded in Table 6, Table 7, Table 8 and Table 9. The term “BoneMarrow dataset” refers to the Hematological Malignancy Bone Marrow Cytology Expert Annotation dataset. The single-category performance analysis across four datasets reveals that ILViT achieves superior metrics on relatively balanced distributions (e.g., WhiteBloodCell), demonstrating robust feature extraction across all categories. However, for datasets with skewed distributions, such as ICPR-HEp-2 and BoneMarrow, the model’s performance drops significantly in minority classes. For example, the Golgi category in ICPR-HEp-2 (recall = 0.7847) and Proerythroblasts in the BoneMarrow (recall = 0.7847) demonstrate significant performance gaps compared to majority classes, despite maintaining high precision. This divergence reflects that the model is more prone to miss-detections (low recall) than misclassifications (high precision). The same phenomenon is also observed in the BioMediTech dataset, further corroborating the impact of data imbalance on model classification performance. Thus, despite the overall excellent performance of ILViT, the data imbalance problem still limits its recognition ability on some categories.

## 5. Discussions and Conclusions

In this study, we propose a novel method, termed ILViT, and evaluate its performance on four public datasets. The model integrates two core components (DIC and COKA) to jointly enable robust classification performance in complex cellular imaging scenarios. The DIC module combines dynamic and Inception-style convolutions, achieving the representational capacity of large-kernel convolutions while maintaining higher efficiency. The COKA module, composed of Omni-dimension dynamic convolution, linear attention, and a KAN, is designed to enhance global feature extraction and nonlinear representation. The ILViT framework first extracts local information via DIC, then iteratively refines this representation with global context through multiple COKA stages. This progressive fusion enables ILViT to surpass the accuracy of current state-of-the-art models in cell classification. In addition, by incorporating quantized components, linear attention, and KAN, the model achieves a lightweight architecture without compromising accuracy.

Experimental results in the BioMediTech, bone marrow, leukocyte classification, and ICPR-HEp-2 datasets (Table 4) confirm the effectiveness of ILViT. Notably, the ILViT model outperforms MViTv2 by 7% in accuracy in the BioMediTech dataset, largely due to the sensitivity of the DIC and COKA modules to both local and global features. Beyond accuracy, ILViT also demonstrates superior efficiency, reducing computational cost and parameter count by 52% and 61%, respectively, compared to MViTv2. To further validate the efficiency of our model, we compared its inference time with the baseline MViTv2 by passing 32 vectors with dimensions (3,224,224) from BioMediTech dataset images to both models simultaneously. The inference time for ILViT is 2.2907 s and that for MViTv2 is 3.0060 s. It implies that our model reduces the inference time by 23.78% over the baseline model. These reductions are primarily attributed to the multi-scale convolution design in DIC and the use of linear attention combined with KAN, which reorganizes matrix operations and approximates complex mappings using combinations of univariate continuous functions. To evaluate model robustness against input noise perturbations, we conduct controlled noise experiments by injecting Gaussian noise (σ = 0.05) into the testing images from the BioMediTech dataset and compare the prediction accuracy before and after noise injection. As shown in Table 10, all models exhibit accuracy drops under noisy conditions. For example, the accuracy of Swin Transformer decreased from 91.11% to 85.80%, Vision Transformer from 90.03% to 88.04%, and ResNet from 91.11% to 80.97%. Similarly, lightweight architectures such as ShuffleNet and RegNet are reduced by 7.23% and 10.69%, respectively. In contrast, our architecture maintains near-identical performance, with its accuracy only decreases from 97.18% to 95.00%, demonstrating superior noise resistance.

Attention visualizations on two datasets further demonstrate that ILViT focuses more precisely on discriminative cellular structures than existing models, highlighting its strong performance and generalizability. Collectively speaking, the results in all four datasets, coupled with the model’s lightweight nature, indicate that ILViT is well suited for a wide range of cell classification tasks. Although the proposed method yields promising results, future work will explore its applicability to broader and more diverse biomedical imaging scenarios to further assess its generalization capability.

In conclusion, this study proposes a lightweight classification method ILViT. The method consists primarily of newly designed DIC and COKA modules. Extensive experiments in four public datasets demonstrate that ILViT achieves excellent feature representation with lower training costs for microscopic cell image classification tasks. We believe that this method holds significant potential for application in a wide range of tasks in various domains. Future work may focus on enhancing the model’s robustness to datasets with unbalanced distributions, integrating with other imaging modalities, and exploring the scalability to larger datasets.

## Figures and Tables

**Table 1:** Four datasets used in the experiments.

Name	Number of Categories	Cell Type	Quantity and Category Names for Each Category
BioMediTech Dataset [25]	4	Retinal Cells	216 images of Fusiform Cells, 547 images of Epithelioid Cells, 949 images of Cobblestone Cells, and 150 images of Mixed Cells
White Blood Cell Dataset [26]	4	Blood Cells	3133 images of Eosinophils, 3108 images of Lymphocytes, 3095 images of Monocytes, and 3171 images of Neutrophils
ICPR-HEp-2 Dataset [27]	6	HEp-2 Cells	2495 images of Homogeneous, 2831 images of Speckled, 2598 images of Nucleolar, 2741 images of Centromere, 2208 images of Nuclear Membrane, and 724 images of Golgi
Hematological Malignancy Bone Marrow Cytology Expert Annotation Dataset [28]	6	Bone Marrow Cells	5883 images of Eosinophils, 3055 images of Degenerative Myelocytes, 4040 images of Monocytes, 6557 images of Bone Marrow Cells, 3538 images of Non-Identifiable Elements, and 2740 images of Proerythroblasts

**Table 2:** Model Training Configuration.

Parameter	Value
OPTIMIZING_METHOD	AdamW
AdamW-eps	$1\times 10^{-8}$
AdamW-betas	(0.9, 0.999)
AdamW-weight_decay	$1\times 10^{-4}$
Batch-size	32
LR	$1\times 10^{-4}$
Lr_scheduler	CosineLRScheduler
Lr_min (lr_scheduler)	$1\times 10^{-6}$
Warmup_lr_init (lr_scheduler)	$1\times 10^{-6}$
Number of Attention Heads	8

**Table 3:** Overall architectures of the comparative models.

Swin Transformer	ViT	ResNet	ConvNeXt	ShuffleNet	RegNet	MViTv2
Conv(4)	Conv(16)	Conv(7)	Conv(4)	Conv(3)	Conv(3)	Conv2d
Stage 1, 2, 3:	Transformer Encoder	BatchNorm2d	LayerNorm	BatchNorm	S1/S2:	LayerNorm
Swin Block × 2	Blocks × 12	ReLU	Conv_layernorm × 3	ReLU	b1:	Pooling Attention
Patch Merging	Multi-head Self-Attention	MaxPool(3)	Conv(2)	InvertedResidual × (1 + 7 + 1 + 3):	Conv(1)	LayerNorm
Stage 4	Linear	BasicBlock × 3	LayerNormBlock × (3 + 3 + 27 + 3):	Conv(1)	Conv(3)	Linear
Swin Block × 2		BasicBlock × 4	DWConv(7)	BatchNorm	Conv(1)	GELU
Global Average Pooling		BasicBlock × 6	LayerNorm	ReLU	Conv(1)	Linear
		BasicBlock × 3	Linear	Linear	S3/S4:	LayerNorm
		AdaptiveAvgPool(1)	GELU		b1:	Linear
		Linear	Linear		Conv(1)	Pooling Attention
			LayerNorm		Conv(3)	LayerNorm
			Linear		Conv(1)	MLP
					Conv(1)	LayerNorm
					b2-b4:	Pooling Attention
					Conv(1)	LayerNorm
					Conv(3)	MLP
					Conv(1)	LayerNorm
					AdaptiveAvg	Pooling Attention
					Pool2d	LayerNorm
					Dropout	MLP
					Linear	LayerNorm
						GlobalAvgPool
						Linear

**Table 4:** Quantitative comparison results of different models on four datasets.

Acc (%)		Dataset	BioMediTech [25]	WhiteBloodCell [26]	ICPR-HEp-2 [27]	BoneMarrow [28]
		
Model						
SwinTransformer [33]			91.11	93.08	95.49	89.09
Vision Transformer [19]			90.03	97.84	96.67	88.14
ResNet [30]			91.11	94.84	96.35	88.45
ConvNext [34]			91.91	94.84	94.70	88.49
ShuffleNet [32]			87.06	93.24	87.46	87.42
RegNet [31]			91.37	95.51	96.06	88.14
MViTv2 [35]			90.49	98.19	95.38	87.21
Ours			97.18	99.75	97.43	90.52

**Table 5:** Ablation experiments on the BioMediTech dataset.

Model	Acc (%)	FLOPs	Params
MViTv2 [35]	90.49	4.00 G	23.33 M
ILViT_V0	94.3182	3.95 G	23.31 M
ILViT_V1	95.8824	3.93 G	23.29 M
ILViT_V2	96.5625	4.22 G	25 M
ILViT_V3	96.7647	4.22 G	25 M
ILViT_V4	97.1875	1.91 G	8.98 M

**Table 6:** Single class performance of ILViT on the BioMediTech dataset.

Class	Accuracy	Precision	Recall
Fusiform	0.9070	0.9512	0.9070
Epithelioid	0.9817	0.9817	0.9817
Cobblestone	0.9947	0.9895	0.9947
Mixed	0.9000	0.8710	0.9000

**Table 7:** Single class performance of ILViT on the WhiteBloodCell dataset.

Class	Accuracy	Precision	Recall
Eosinophils	0.9952	0.9984	0.9952
Lymphocytes	0.9968	0.9984	0.9968
Monocytes	0.9984	0.9952	0.9984
Neutrophils	0.9984	0.9969	0.9984

**Table 8:** Single class performance of ILViT on the ICPR-HEp-2 dataset.

Class	Accuracy	Precision	Recall
Homogeneous	0.9840	0.9609	0.9840
Speckled	0.9876	0.9739	0.9876
Nucleolar	0.9846	0.9808	0.9846
Centromere	0.9872	0.9872	0.9872
Nuclear Membrane	0.9796	0.9664	0.9796
Golgi	0.7847	0.9741	0.7847

**Table 9:** Single class performance of ILViT on the BoneMarrow dataset.

Class	Accuracy	Precision	Recall
Eosinophils	0.9549	0.9212	0.9549
Degenerative Myelocytes	0.8314	0.8684	0.8314
Monocytes	0.9035	0.8979	0.9035
Bone Marrow Cells	0.9634	0.9321	0.9634
Non-Identifiable Elements	0.8741	0.8944	0.8741
Proerythroblasts	0.7847	0.8635	0.7847

**Table 10:** Robustness evaluation of different models against Gaussian noise on the BioMediTech dataset.

Dataset	SwinTransformer	ViT	ResNet	ConvNext	ShuffleNet	RegNet	MViTv2	Ours
With Gaussian Noise	85.80	88.04	80.97	81.25	79.83	80.68	87.78	95.00
Original	91.11	90.03	91.11	91.91	87.06	91.37	90.49	97.18

## Data Availability

The data presented in this study are openly available in Figshare at 10.1371/journal.pone.0149399, reference number [25], Kaggle at https://www.kaggle.com/datasets/paultimothymooney/blood-cells, reference number [26], ModelWhale at https://www.heywhale.com/mw/dataset/5ec3c6883241a100378d5d4a, reference number [27], Wiki at 10.1182/blood.2020010568, reference number [28], respectively.
